# Effects of Immunoglobulins G From Systemic Sclerosis Patients in Normal Dermal Fibroblasts: A Multi-Omics Study

**DOI:** 10.3389/fimmu.2022.904631

**Published:** 2022-06-29

**Authors:** Aurélien Chepy, Solange Vivier, Fabrice Bray, Camille Ternynck, Jean-Pascal Meneboo, Martin Figeac, Alexandre Filiot, Lucile Guilbert, Manel Jendoubi, Christian Rolando, David Launay, Sylvain Dubucquoi, Guillemette Marot, Vincent Sobanski

**Affiliations:** ^1^Univ. Lille, Inserm, CHU Lille, U1286 - INFINITE ( Institute for Translational Research) in Inflammation, Lille, France; ^2^CHU Lille, Département de Médecine Interne et Immunologie Clinique, Centre de Référence des Maladies Auto-immunes Systémiques Rares du Nord et Nord-Ouest de France, Lille, France; ^3^Univ. Lille, CNRS, USR 3290, Miniaturisation pour la Synthèse, l’Analyse et la Protéomique, Lille, France; ^4^Univ. Lille, CHU Lille, ULR 2694, METRICS: Évaluation des Technologies de Santé et des Pratiques Médicales, Lille, France; ^5^Univ. Lille, CNRS, INSERM, CHU Lille, Institut Pasteur de Lille, US 41—UAR 2014-PLBS, Lille, France; ^6^CHU Lille, Institut d’Immunologie, Lille, France; ^7^Inria, Models for Data Analysis and Learning, Lille, France; ^8^Institut Universitaire de France, Paris, France

**Keywords:** systemic sclerosis, multi-omics analysis, autoantibodies, proteomics, transcriptomics

## Abstract

Autoantibodies (Aabs) are frequent in systemic sclerosis (SSc). Although recognized as potent biomarkers, their pathogenic role is debated. This study explored the effect of purified immunoglobulin G (IgG) from SSc patients on protein and mRNA expression of dermal fibroblasts (FBs) using an innovative multi-omics approach. Dermal FBs were cultured in the presence of sera or purified IgG from patients with diffuse cutaneous SSc (dcSSc), limited cutaneous SSc or healthy controls (HCs). The FB proteome and transcriptome were explored using liquid chromatography coupled with tandem mass spectrometry (LC-MS/MS) and microarray assays, respectively. Proteomic analysis identified 3,310 proteins. SSc sera and purified IgG induced singular protein profile patterns. These FB proteome changes depended on the Aab serotype, with a singular effect observed with purified IgG from anti-topoisomerase-I autoantibody (ATA) positive patients compared to HC or other SSc serotypes. IgG from ATA positive SSc patients induced enrichment in proteins involved in focal adhesion, cadherin binding, cytosolic part, or lytic vacuole. Multi-omics analysis was performed in two ways: first by restricting the analysis of the transcriptomic data to differentially expressed proteins; and secondly, by performing a global statistical analysis integrating proteomics and transcriptomics. Transcriptomic analysis distinguished 764 differentially expressed genes and revealed that IgG from dcSSc can induce extracellular matrix (ECM) remodeling changes in gene expression profiles in FB. Global statistical analysis integrating proteomics and transcriptomics confirmed that IgG from SSc can induce ECM remodeling and activate FB profiles. This effect depended on the serotype of the patient, suggesting that SSc Aab might play a pathogenic role in some SSc subsets.

## Introduction

Systemic sclerosis (SSc) is a connective tissue disease characterized by extensive fibrosis of the skin and internal organs, associated with vasculopathy and autoimmune features (1). The pathophysiology of SSc is characterized by a triad including autoimmunity, vasculopathy, and excessive fibrosis due to increased extracellular matrix (ECM) synthesis by activated fibroblasts (FB). The humoral immune system plays an important role in the pathophysiology of SSc (2). Autoantibodies (Aabs) are found and used in almost all patients. Some of these anti-nuclear Aabs are assessed in routine clinical practice and are robust diagnostic and prognostic biomarkers. For example, anti-topoisomerase-I autoantibodies (ATAs) are usually associated with dcSSc and the presence and severity of interstitial lung disease, whereas anticentromere autoantibodies (ACAs) are associated with lcSSc (3). The potential pathogenic role of these Aabs remains elusive as they target intracellular antigens, but some recent studies have suggested an indirect direct role in FB and endothelial cells *via* immune complexes (4–6). Other Aabs other than those used in daily clinical practice may also play a direct role in the pathophysiology of SSc, such as anti-fibroblast Aabs (7).

However, the link between Aabs and fibrosis during SSc remains largely unexplained. A few studies in the literature have attempted to explain this link, most often using targeted approaches (thus limiting the number of exploitable biological processes) or focusing on a particular Aab. Multi-omics approaches allow a global and unbiased analysis of gene or protein expression and can improve our knowledge of disease mechanisms (8, 9). This study explored the effect of purified IgG from SSc patients on the phenotype and function of normal dermal FB using an innovative multi-omics approach combining proteomics by liquid chromatography coupled with tandem mass spectrometry (LC-MS/MS) and transcriptomics using DNA microarrays.

## Materials and Methods

### Population

Sera from 5 ATA positive (ATA+) dcSSc patients, 5 ATA negative (ATA−) dcSSc patients, and 5 ACA positive (ACA+) lcSSc patients from the Internal Medicine Department of the Lille University Hospital (CHU) were prospectively collected in a declared biobank (CPP no. 2019-A01083-54, CNIL Data Protection Officer No. 2018_82/DEC19-553, NCT04334031) and retrieved for this study (**Supplemental Tables 1****,** **2**) along with those from 5 healthy controls (HC).

IgG was purified from the sera using protein G columns: the NAbProtein G spin kit (Thermo Fischer Scientific). For each individual, total sera and purified IgG were then obtained and stored at −80°C before experiments. Briefly, each serum was processed in a protein G affinity column. After the first centrifugation step, the IgG-depleted serum was collected. Then, after a second centrifugation step, the purified IgG fractions were collected in a phosphate-saline solution. A total IgG-level measurement and serotype assay were performed to ensure the validity of the purification step. IL-10, IL-1β, IL-6, and TNF-α were measured in 2 dcSSc ATA+ purified IgG samples with Ella Automated Immunoassay Systems, R&D Systems (**Supplemental Table 3**).

### Cell Culture

Primary dermal human FB (ATCC^®^ Number: PCS-201-012 ™) were cultured at the 5th passage in the presence of sera or purified IgG. FB were initially cultured with Dulbecco’s Modified Eagle medium combined with antibiotics at 1% concentration (penicillin and streptomycin) and 10% fetal calf serum (FCS). To avoid interference due to the presence of FCS, cells were FCS-deprived for 24 h after seeding. For proteomic analysis and immunofluorescence, FBs were cultured for 72 h in the presence of both sera and purified IgG at a concentration of 100 µg/ml or TGF-β1 at a concentration of 5 ng/ml. The IgG concentration was normalized at 100 µg/ml of IgG for both sera and purified IgG. For each condition, the volume of serum was added to obtain an IgG concentration of 100 µg/ml. For transcriptomic analysis, FB were cultured for 24 h in the presence of purified IgG at a normalized concentration of 100 µg/ml. The purified IgG samples were mostly composed of IgG and proinflammatory cytokines such as IL-10, IL-1β, and IL-6 of TNF-α were below or at the detection limit threshold (**Supplemental Table 3**).

### Proteomics

Proteins from cell culture were extracted, then digested using the eFASP method and quantified with the label-free method by LC-MS/MS Orbitrap (Thermo Scientific; Q Exactive Plus) (10). Each sample was separated by a C18 column on nanoliquid chromatography (Thermo Scientific; U3000 RSLC). The analysis of the raw LC-MS/MS data was performed using MaxQuant (version 1.5.2.8) and the Andromeda search engine. The database supplied to the search engine was a UniProtKB/Swiss-Prot human protein database (2021-03, *Homo sapiens*, Sequences: 75,074). For a more precise label-free quantification of proteins, MaxQuant uses a dedicated algorithm called MaxLFQ for intensity determination. Normalization was performed using the Perseus software, after selecting proteins with 2 out of 5 valid values for each condition (dcSSc ATA+, dcSSc ATA−, lcSSc ACA+ and HC). Data were log2-tranformed and missing values were imputed with a normal distribution (11). The mass spectrometry proteomic data have been deposited on the Proteome Xchange Consortium *via* the PRIDE partner repository with the dataset identifier PXD025885.

### Transcriptomics

Total ribonucleic acid (RNA) from cells in the presence of purified IgG from dcSSc and HC was prepared using the RNeasy mini-kit (QIAGEN) according to the instructions of the manufacturer, including the additional step of DNase treatment. Total RNA yield and quality were further assessed on the Agilent 2100 bioanalyzer (Agilent Technologies). One-color whole human (072363_D_F_20190204 slides) 60-mer oligonucleotides 8 × 60 k microarrays (Agilent Technologies) were used to analyze gene expression. Complementary RNA (cRNA) labeling, hybridization, and detection were carried out according to the instructions of the supplier (Agilent Technologies). For each microarray, cyanine 3-labeled cRNA was synthesized with the low input QuickAmp labeling kit from 50 ng of total RNA. RNA Spike-In was added to all tubes and used as a positive control for labeling and amplification steps. The labeled cRNA was purified and 600 ng of each cRNA was then hybridized and washed following the instructions of the manufacturer. Microarrays were scanned on an Agilent G2505C scanner and data were extracted using Agilent Feature Extraction Software^©^ (FE version 10.7.3.1).

Data were log2-transformed and values higher than a background threshold based on the 95th percentile of negative controls in all biological replicates of at least one condition were kept. Microarray data are available through the GEO depository from NCBI: GSE178299.

### Differential Analyses

Differential analyses were performed using the Bioconductor R package *Limma* (version 3.38.3) (12). Raw p-values were adjusted with the Benjamini–Hochberg method (13). In proteomics, differentially expressed proteins (DEP) were defined for each comparison using cut-offs of adjusted p-value <0.05 and log2 fold change (LogFC) >1.4 or <−1.4. **Table 1** presents the number of DEP in each condition. In transcriptomics, differentially expressed genes (DEG) were defined for each comparison using cut-offs of adjusted p-value <0.1 and fold change (FC) >1.5 or <−1.5. Venn diagrams were generated using VENNY (14).

**Table 1:** Comparisons considered for proteomic analysis and differentially expressed proteins.

Comparisons	Numbers of differentially expressed proteins
[SSc] [IgG] vs [HC] [IgG]	54
[SSc] [Sera] vs [HC] [sera]	28
[SSc] [Sera] vs [SSc] [IgG]	255
[SSc] [IgG] [ATA+] vs [HC] [IgG]	629
[SSc] [IgG] [ATA+] vs [SSc] [IgG] [ATA-]	372
[SSc] [IgG] [ATA+] vs [SSc] [IgG] [ACA+]	258
[SSc] [IgG] [ATA-] vs [HC] [IgG]	0
[SSc] [IgG] [ACA+] vs [HC] [IgG]	1

[HC] [IgG], IgG from healthy controls; [SSc] [Sera], sera from SSc (all serotypes); [SSc] [IgG], IgG from SSc (all serotypes); [SSc] [IgG] [ATA+], IgG from dcSSc anti-topoisomerase-I positive patients; [SSc] [IgG] [ATA−], IgG from dcSSc anti-topoisomerase-I negative patients; [SSc] [IgG] [ACA+], IgG from lcSSc anti-centromere positive patients.

### Visualization of Identified Proteins and Genes of Interest

The visual exploration was carried out with principal component analyses (PCA) either on identified proteins or on specific genes of interest. These genes of interest were defined as the genes corresponding to DEP between IgG ATA+ and IgG HC. Heatmaps were plotted using the *pheatmap* R package. Additionally, a cluster analysis was carried out with an ascendant hierarchical clustering.

### Functional Enrichment

Functional enrichment and pathway analysis were conducted using Metascape (https://metascape.org/gp/index.html) with the Gene Ontology terms (GO terms) and Hallmark gene sets. Metascape uses the hypergeometric distribution (Fisher’s exact test) to identify all ontology terms that contain a statistically greater number of candidates in common with an input list than expected by chance. As recommended in Metascape guidelines, terms with a *p*-value <0.01, a minimum of 3 candidates, and an enrichment factor >1.5 were selected for visualization (15).

### Proteomics and Transcriptomics Data Integration

The whole transcriptomics and proteomics datasets were integrated using the DIABLO (Data Integration Analysis for Biomarker discovery using Latent cOmponent) R mixOmics framework (16, 17). Nine proteins without a gene name were excluded from integration analysis. This sparse multivariate approach maximizes both the correlations between the omics datasets and the discriminative ability of the resulting model on the groups studied here (dcSSc ATA+, dcSSc ATA− or HC). DIABLO thus highlights co-expressed genes and proteins associated with the group variable in a supervised and parsimonious fashion. In our study, the non-diagonal coefficients of the design matrix were set to 0.9. This prioritizes the exploration of meaningful correlations between selected genes, proteins, and the group, rather than predicting the group itself. Given the small number of samples (n = 15), the DIABLO algorithm was thus employed from an exploratory perspective rather than a predictive one. Cross-validation (50 × 5-fold) was used to choose the number of latent components (here 2) and the number of variables per component. From two input datasets composed of 24,490 transcripts and 3,301 proteins, respectively, 50 and 5 genes were kept to derive the 2 transcriptomics-based latent components, whereas 80 and 50 proteins were retained to derive the 2 proteomics-based latent components.

### Immunofluorescence Assays

After culture (same conditions as for proteomics), FB were fixed in paraformaldehyde 4% and permeabilized with triton 0.05% and then 0.1%. FB were saturated with PBS/bovine serum albumin 1% for 30 min, reducing the unspecific binding of antibodies to non-target structures. Primary and secondary antibodies were diluted in a PBS/BSA 1% solution. Primary antibodies were incubated over night after a washing step with PBS/triton 0.05%. Secondary antibodies were incubated for 1 h. After the secondary antibody incubation step, the slats were mounted on a microscope slide using mounting medium containing DAPI for nuclei staining (Sigma-Aldrich, DUO82040). Microscope slides were read using a LEICA DMI8 microscope and images were analyzed with ZEN Blue Edition 3.3 software (Zeiss). Details for primary and secondary antibody references and the dilution used are available in **Supplementary Methods**.

## Results

### Sera and Purified IgG From SSc Patients Modify the Proteome of FB in a Serotype-Dependent Manner

**Figure 1:**
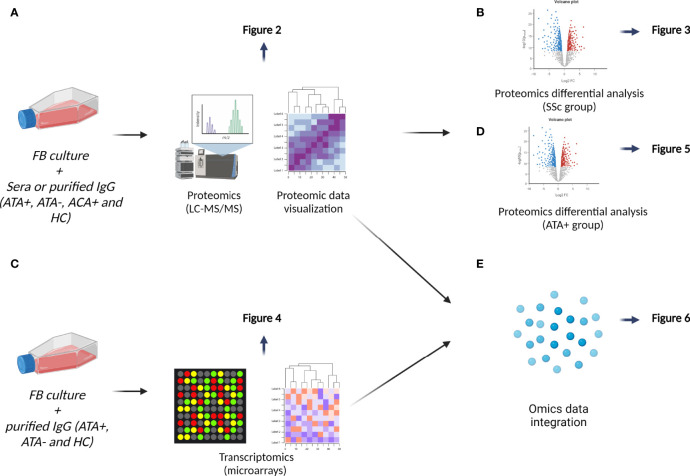
Data analysis workflow. **(A)** We first performed principal component analysis on proteomics data and then hierarchical clustering results were visualized on heatmap, see **Figure 2**. **(B)** We explored effect of SSc sera and SSc IgG (all serotypes) by conducting differential analysis, see in **Figure 3**. **(C)** Transcriptomic analyses of FB cultured in the presence of IgG from dcSSc (ATA+ and ATA−) and HC were conducted by proteomics results, see in **Figure 4**. **(D)** We then focused on the singular group “purified IgG from ATA+ patients” in proteomics, see in **Figure 5**. **(E)** Finally, we performed a second way of multi-omics integration from whole proteomics and transcriptomics data, see in **Figure 6**.

**Figure 2:**
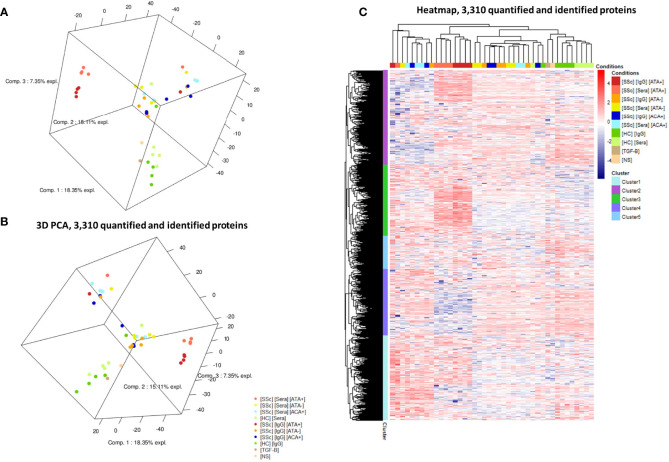
Protein expression profiles in LC-MS/MS analysis. **(A, B)**: two different views of 3D-PCA scatter plots for the analyzed cell samples. PCA highlighted FB protein expression according to the patient serotype of the purified IgG. **(C)** Heatmap representing the 3,310 differentially expressed proteins in all samples. Cluster analysis identified 5 different clusters of protein expression. [HC] [Sera], sera from healthy controls; [HC] [IgG], IgG from healthy controls; [SSc] [Sera] [ATA+], sera from dcSSc anti-topoisomerase-I positive patients; [SSc] [IgG] [ATA+], IgG from dcSSc anti-topoisomerase-I positive patients; [SSc] [Sera] [ATA−], sera from dcSSc anti-topoisomerase-I negative patients; [SSc] [IgG] [ATA−], IgG from dcSSc anti-topoisomerase-I negative patients; [SSc] [Sera] [ACA+], from sera lcSSc anti-centromere positive patients; [SSc] [IgG] [ACA+], IgG from lcSSc anti-centromere patients; [TGF-B], FB stimulated in the presence of TGF-β; [NS], non stimulated FB. PCA, principal component analysis.

**Figure 3:**
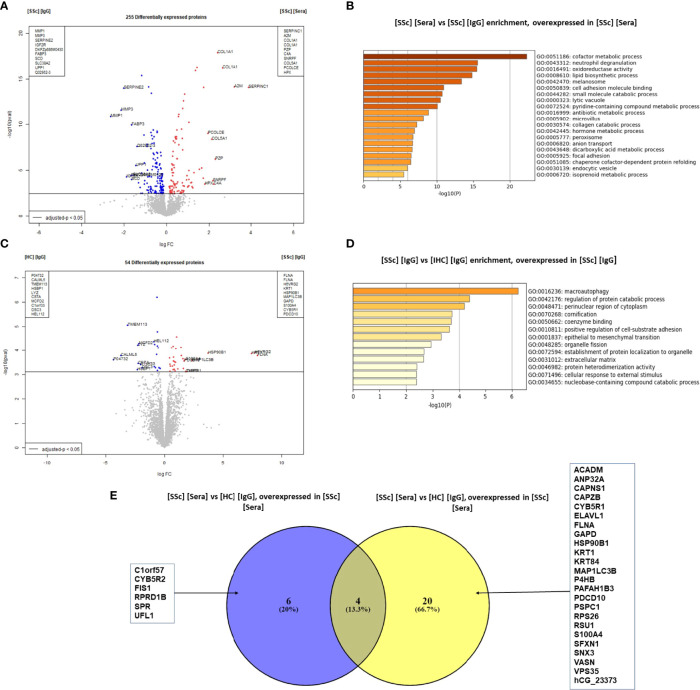
SSc purified IgG induced ECM remodeling profile in FB at the protein level. **(A)** "volcano plot" representing the comparison [SSc] [IgG] vs [SSc] [Sera]; **(B)** Enriched clusters GO terms in the comparison [SSc] [IgG] vs [SSc] [Sera] according to upregulated proteins (overexpressed in [SSc] [sera]); **(C)** volcano plot representing the comparison [SSc] [IgG] vs [HC] [IgG]; **(D)** Enriched clusters GO terms in the comparison [SSc] [IgG] vs [HC] [Sera] according to upregulated proteins (overexpressed in [SSc] [IgG]); **(E)** Venn diagram representing commonly or exclusively overexpressed proteins in the comparison [SSc] [sera] vs [HC] [Sera]. Proteins are shown by their gene names. Proteins with adjusted p-value < 0.05 and Log Fold Change >1.4 or <−1.4 were considered for each comparison (volcano plot). Enriched clusters GO terms and network were obtained using Metascape. [SSc] [sera], sera from SSc; [SSc] [IgG], IgG from SSc; [HC] [IgG], IgG from heathy controls; [SSc] [IgG] [ATA+], IgG from dcSSc anti-topoisomerase-I positive patients; [SSc] [IgG] [ATA−], IgG from dcSSc anti-topoisomerase-I negative patients; [SSc] [IgG] [ACA+], IgG from lcSSc anti-centromere positive patients; GO terms, gene ontology terms.

**Figure 4:**
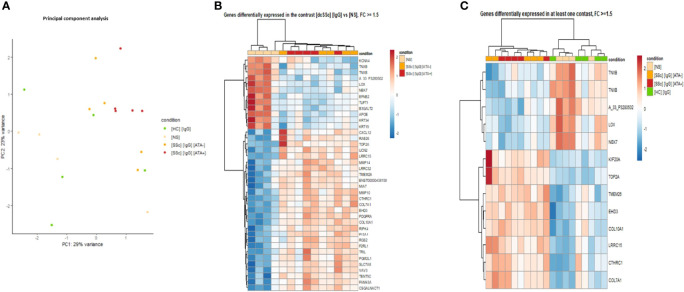
Transcriptomics identified FB ECM remodeling changes induced by dcSSc-purified IgG. **(A)** PCA representing the 629 DNA probes (transcriptomics) dysregulated in proteomic analysis highlights genes expression by the FB according to SSc serotype. Purified IgG from ATA+ patients induced a singular gene expression profile compared to ATA− SSc patients and HC; **(B)** Heatmap representing genes dysregulated in the contrast [dcSSc] [IgG] vs [NS] with FC >1.5; **(C)** Heatmap representing top-ranked genes dysregulated with FC >1.5. FC, fold change; [dcSSc] [IgG], IgG from dcSSc (anti-topoisomerase-I positive and anti-topoisomerase-I negative patients); [SSc] [IgG] [ATA+], IgG from dcSSc anti-topoisomerase-I positive patients; [SSc] [IgG]; [ATA−], IgG from dcSSc anti-topoisomerase-I negative patients; [NS], non stimulated fibroblasts; [HC] [IgG], IgG from healthy controls.

**Figure 5:**
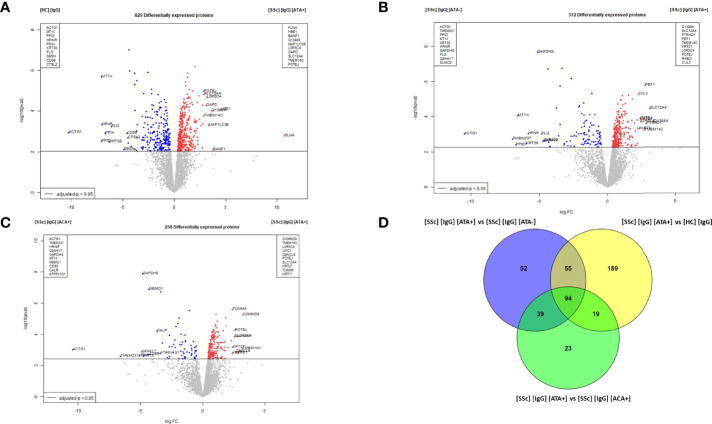
Proteomic analysis revealed that dcSSc ATA+ purified IgG induced singular modifications in normal dermal FB. **(A)** volcano plot representing the comparison [SSc] [IgG] [ATA+] vs [HC] [IgG]; **(B)** volcano plot representing the comparison [SSc] [IgG] [ATA+] vs [SSc] [IgG] [ATA−]; **(C)** volcano plot representing the comparison [SSc] [IgG] [ATA+] vs [SSc] [IgG] [ACA+]; **(D)** Venn diagram representing commonly overexpressed proteins in [IgG] [ATA+] vs other groups. Proteins with adjusted p-value <0.05 and Log Fold Change >1.4 or <−1.4 were considered for each comparison (Volcanoplot). Enriched clusters GO terms and network were obtained using Metascape. [SSc] [sera], sera from SSc; [SSc] [IgG], IgG from SSc; [HC] [IgG], IgG from heathy controls; [SSc] [IgG] [ATA+], IgG from dcSSc anti-topoisomerase-I positive patients; [SSc] [IgG] [ATA−], IgG from dcSSc anti-topoisomerase-I negative patients; [SSc] [IgG] [ACA+], IgG from lcSSc anti-centromere positive patients.

We first analyzed the FB proteome in the presence of sera and purified IgG from SSc patients (**Figure 1**). Proteomics by LC-MS/MS analysis identified and quantified 3,310 proteins. Comparing SSc vs. HC, we identified 28 DEP using sera and 54 DEP using purified IgG (**Table 1**). Proteomic profiles were distinct between the SSc and HC groups. PCA identified 4 groups of subjects according to the induced protein expression patterns in FB. Two were homogeneous: the first including only dcSSc ATA+ patients and the second including only HC. The two other groups were more heterogeneous: the third included mostly dcSSc ATA− patients, and the fourth group included mainly lcSSc ACA+. The protein expression profiles of the second and fourth groups seemed rather different from those of the first and third groups (**Figures 2A, B**). A 3D visualization of PCA is available online (**Supplementary Appendix, Video**). Hierarchical clustering analysis of the 3,310 identified proteins showed 5 distinct clusters (**Figure 2C**). The first cluster included 803 proteins, mostly over-expressed in the ACA+ group. Proteins of the enriched GO terms were involved in the nucleotide metabolic process, focal adhesion, or actin cytoskeleton. The second cluster included 894 proteins, mostly over-expressed in the ATA+ group. Proteins of the enriched GO terms were involved in cellular response to stress and vesicle-mediated transport. The third cluster included 673 proteins, mostly over-expressed in the ATA+ group. Proteins of the enriched GO terms were involved in translation, focal adhesion, or macroautophagy. The fourth cluster included 626 proteins, mostly under-expressed in the ATA+ group. Enriched GO terms were nucleotide metabolic processes and organic cyclic compound catabolic processes. Finally, the fifth cluster included 314 proteins. Enriched GO terms were nucleotide metabolism, focal adhesion, and regulation of cellular protein localization (**Supplemental Appendix, Figures 1–5**).

### SSc Purified IgG Induced ECM Remodeling Profile in FB at the Protein Level

We then compared the effects of SSc sera and purified IgG on FB. The comparison of the FB proteome in the presence of SSc sera vs. SSc-purified IgG showed 255 DEP (**Figure 3A**). ECM proteins like type 1- and type 3-collagens were overexpressed in the presence of SSc sera (LogFC: 2.4; adjusted p-value <0.0001 and LogFC: 1.7; adjusted p-value <0.0001; respectively). Enriched GO terms in the SSc sera group were: cofactor metabolic process, cell adhesion molecule binding, and collagen catabolic process (**Figure 3B**).

The comparison of the FB proteome in the presence of purified IgG from SSc vs. HC revealed 54 DEP. Of these, filamin A was the most strongly associated protein with SSc-purified IgG (LogFC: 10.9 and adjusted p-value: 0.00085). Other proteins like S100A4 were also overexpressed (LogFC: 1.8 and adjusted p-value: 0.013) (**Figure 3C**). Using immunofluorescence, we confirmed the overexpression of S100A4 in FB exposed to SSc-purified IgG, especially pronounced in the ATA+ group (**Supplemental Figure 6A**). Enriched GO terms in the SSc-purified IgG group were macroautophagy, regulation of protein catabolic processes, and the perinuclear region of the cytoplasm (**Figure 3D**). Among the 24 proteins overexpressed in the SSc-purified IgG group vs. HC-purified IgG group, only 4 were also overexpressed in the SSc sera group vs. HC sera group comparison (**Figure 3E**).

### Transcriptomics Identified FB Extracellular Matrix Remodeling Changes Induced by dcSSc Purified IgG

First, to strengthen our previous findings and integrate multi-omics data, transcriptomic analyses were driven by proteomic results (**Figure 1C**). PCA was performed by restricting transcriptomic analysis on all genes of DEP identified in proteomics with |FC| >1.5 in at least one contrast and confirmed the singular group of FB exposed to dcSSc ATA+ purified IgG (**Figure 4A**).

Then, we performed DEG analysis. The comparison of FB exposed to dcSSc-purified IgG vs. non-stimulated FB identified 764 DEG, of which 455 were upregulated in FB exposed to dcSSc purified IgG. By applying a cut-off of |FC| >1.5, we identified 39 DEG, of which 27 were upregulated in FB exposed to dcSSc-purified IgG. Cluster analysis of those 39 DEG found two groups of gene expression. Genes like type 7- and 10-collagen, collagen triple helix repeat-containing protein 1, platelet-derived growth factor receptor alpha, matrix metallopeptidase-10 and 14 were upregulated in a first cluster containing FB in the presence of dcSSc-purified IgG (**Figure 4B**).

Furthermore, using the top-ranked DEG identified and visualizing them on a heatmap displaying all samples, we were able to identify 2 distinct clusters of gene expression. A first cluster included genes overexpressed in the FB exposed to the dcSSc-purified IgG group (mitotic kinesin, type 2A topoisomerase, transmembrane protein 26, eps15 homology domain-containing protein 3, type 1 and 7 collagen, collagen triple helix repeat-containing protein 1, and leucine rich repeat containing 15). The second cluster included genes overexpressed in FB exposed to HC-purified IgG and non-stimulated FB (tenascin XB, lysyl oxidase, and serine/threonine-protein kinase nek7) (**Figure 4C**).

### Proteomics Analysis Revealed That dcSSc ATA+ Purified IgG Induced Singular Modifications in Normal Dermal FB

To confirm the singular effect of dcSSc ATA+ purified IgG, we compared FB exposed to dcSSc ATA+ purified IgG vs. HC purified IgG and dcSSc ATA+ purified IgG vs. dcSSc ATA− purified IgG (**Figure 1D**).

Six hundred and twenty-nine DEP were identified in the dcSSc ATA+ purified IgG vs. HC-purified IgG comparison. Among them, filamin A, proteins like microtubule-associated proteins 1A/1B light chain3 B (MAP1LC3B), solute carrier family 12, or member or transmembrane protein 14C were overexpressed in FB in the presence of dcSSc ATA+ purified IgG. Of these, filamin A was the most strongly associated with the presence of IgG from dcSSc ATA+ compared to IgG from HC (LogFC: 10.8 and adjusted p-value: 0.0194) (**Figure 5A**). Enriched GO terms in the dcSSc ATA+ purified IgG group compared to HC-purified IgG were involved in focal adhesion, translation, cell adhesion molecule binding, or actin binding (**Supplemental Figure 7A**). Three hundred and seventy-two DEP were identified in FB in the presence of dcSSc ATA+ purified IgG vs. dcSSc ATA− purified IgG. Among them, ras-related protein 21, solute carrier family 12, member 4 transmembrane protein 14C, or cullin-3 were overexpressed in FB in the presence of ATA+ purified IgG (**Figure 5B**). Using immunofluorescence, we confirmed the overexpression of ras-related protein 21 in FB exposed to SSc-purified IgG from ATA+ patients (**Supplemental Figure 6B**). Enriched GO terms in the dcSSc ATA+ purified IgG group were involved in focal adhesion, cadherin binding, cytosolic part, or lytic vacuole (**Supplemental Figure 7B**). Two hundred and fifty-eight DEP were identified in dcSSc ATA+ purified IgG vs. lcSSc ACA+ purified IgG comparison. Among them, POTE ankyrin domain family member J, transmembrane protein 14C, or solute carrier family 12 member 4 were overexpressed in FB in the presence of ATA+ purified IgG (**Figure 5C**). Enriched GO terms in the dcSSc ATA+ purified IgG group were involved in the nucleosome, secretory granule lumen, and focal adhesion (**Supplemental Figure 7C**). Finally, 94 proteins were commonly overexpressed in the presence of dcSSc ATA+ purified IgG group such as transgelin, myosin light chain 9 or tubulin beta 1 class VI (**Figure 5D** and **Supplemental Table 4**). Enriched GO terms of these commonly overexpressed proteins were involved in cytoplasmic translation and cadherin binding (**Supplemental Figure 7D**).

### Transcriptomics and Proteomics Data Integration Confirmed Specific Biological Modifications of FB in the Presence of Purified IgG From dcSSc ATA+ and ATA− Patients

**Figure 6:**
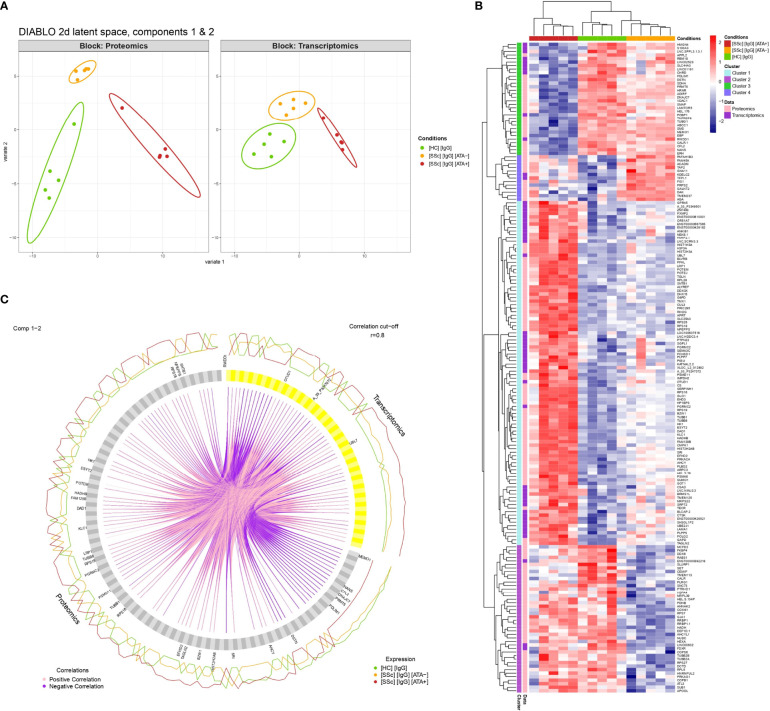
Multi-omics data integration. **(A)** Individual plots representing patients’ projections into the 2D proteomics and transcriptomics space. X and Y-axes correspond to the first and second latent components of the DIABLO algorithm related to either proteomics (Block: Proteomics) or transcriptomics (Block: Transcriptomics) variables. **(B)** Circle plot representing variables with positive and negative correlation between proteomics and transcriptomics with r > 0.8. Only variables with r > 0.9 are tagged. **(C)** Heatmap representing clustering results of multi-omics signature identified by the DIABLO algorithm. [SSc] [IgG] [ATA+], IgG from dcSSc anti-topoisomerase-I positive patients; [SSc] [IgG] [ATA−]: IgG from dcSSc anti-topoisomerase-I negative patients; [SSc] [IgG] [ACA+], IgG from dcSSc anti-topoisomerase-I negative patients; [HC] [IgG], IgG from healthy controls.

Sample plots of the DIABLO model highlighted the excellent discrimination of the 3 groups (dcSSc ATA+ patients, dcSSc ATA− patients, and HC) for proteomics and transcriptomics (**Figures 1E**, **6A**). Selected proteomics and transcriptomics variables and their contributions to each latent component are given in **Supplemental Figures 8, 9**. For example, basic leucine zipper and W2 domains 1 or LDL receptor-related protein 1 contributed the most to the singularity of the ATA+ group in proteomics, whereas protein arginine methyltransferase 5 contributed the most to the singularity of the HC group (**Supplemental Figures 8, 9**). The links between these selected omics variables indicate strong positive or negative correlation and are represented on a circle plot (**Figure 6C**). For example, tubulin beta-1 was overexpressed on proteins in the ATA+ group and correlated positively with transcripts. The heatmap related to clustering performed on the DIABLO omics signature highlighted 4 clusters of variables (combining proteomics and transcriptomics) discriminating the 3 groups (dcSSc ATA+ patients, dcSSc ATA− patients, and HC). The dcSSc ATA+ group appeared more distinct. Cluster 1 was composed of 97 variables, mostly overexpressed in the dcSSc ATA+ group (**Figure 6B**). These variables were enriched in small ribosomal submit and cadherin binding (**Supplemental Figure 10**). Thirty-one of these variables were in common with overexpressed proteins in purified IgG from dcSSc ATA+ in proteomics, among which transgelin and tubulin beta 1 class VI (**Supplemental Table 5**).

## Discussion

This multi-omics study highlighted that sera and purified IgG from SSc modified the proteome and transcriptome profiles of normal dermal FB in a serotype-dependent manner, i.e., depending on the SSc-associated Aabs of patients. DcSSc-purified IgG induced ECM remodeling and activated profiles on dermal FB with a singular and specific effect in ATA+ patients.

Aabs are detected in about 95% of SSc patients and are associated with distinct disease subtypes and organ involvement. Most of them have nuclear targets and are SSc-specific (3). Some studies suggested that IgG ATA+ titers correlate with severity, and recent data showed that IgM ATA+ titers are associated with disease activity (17, 18). Finally, autoantibodies have been found in the sera of individuals years before SSc onset (19). Taken together, these data highlight the potential contribution of Aabs to the onset and/or perpetuation of the disease, suggesting a potential pathogenic role.

Functional antibodies have been described as playing a direct role in the pathophysiology of SSc (7). These Aabs are targeted against the following: a) cell types like anti-endothelial or fibroblasts; b) membranous receptors like angiotensin II type 1 receptor, endothelin-1 type A receptor, or Platelet-Derived Growth Factor receptor; and c) ECM components like fibrillin or matrix metalloproteinase-1 (20–25). Nevertheless, these Aabs are not evaluated in daily practice and do not seem entirely SSc-specific. Thus, evidence of the pathogenic role of anti-nuclear Aab is lacking. Recent data highlight that immune complexes may induce ECM remodeling changes in FB through endothelial damage and that commercial ATA polyclonal IgG induces overexpression of ACTA2 and COL1A1 in FB from SSc (5, 6). However, the potential direct pathogenic role of anti-nuclear Aabs remains rarely studied and is unproven in SSc.

High-throughput technologies such as multi-omics seem to be a promising tool for elucidating key pathogenic processes in SSc by studying skin fibrosis and peripheral blood mononuclear cells (26, 27). Many studies have focused on transcriptomic analysis of SSc skin, contributing to deciphering the clinical and molecular heterogeneity of the disease. Comparison of gene expression in skin and esophageal biopsies identified 4 main molecular SSc subsets: fibroproliferative, inflammatory, limited, and normal-like (28–30). Moreover, by studying transcriptomic profiles of SSc skin, Imano et al. showed that some gene expression and pathways were shared among the different SSc serotypes, whereas others were specific (31). Sensitive proteomics by LC-MS/MS allowed better characterization of the role of plasma B-cells in lung fibrosis (32). To our knowledge, our study is the first to assess the effect of purified IgG on FB in a combined approach with sensitive proteomic and transcriptomic approaches.

Proteomic analysis revealed that SSc sera and purified IgG induced different protein expressions according to the serotype of Aabs present in patients. Visualization of proteomics data identified two main subsets: sera and purified IgG from dcSSc ATA+ patients and HC. These groups were not explained by single sample-related variability. The most important between-group variability was observed between the ATA+ and HC groups.

SSc sera appeared to have profibrotic properties on FB like collagen production. Sera contain IgG. Nevertheless, by studying the SSc-purified IgG separately, a singular biological effect was also highlighted. We observed that the protein expression of FB exposed to SSc-purified IgG was mainly enriched in macroautophagic processes. Autophagy is an essential intracellular self-degradation system and is involved in SSc physiopathology as shown by the loci of the involved gene in a genome-wide association study (33). In our study, the microtubule-associated protein 1A/1B light chain 3B (MAP1LC3B), which is a marker of the autophagosome, was overexpressed. This protein was also expressed in the skin of an SSc mouse model and patients (34). Moreover, ras-related protein 21 (a protein involved in autophagosome–lysosome fusion) was overexpressed under dcSSc ATA+ purified IgG conditions (35).

The ATA+ group appeared the most different compared with HC. Moreover, there was no DEP in the comparison between ATA− vs HC and ACA+ vs HC. Within this particular ATA+ group, DEP were enriched in focal adhesion and cadherin-binding GO terms. Proteins involved in focal adhesion GO terms serve a structural role, linking the ECM from the outside to the actin cytoskeleton on the inside of the cell (36). Cadherin-binding participates in cell adhesion. Of this cadherin binding family, cadherin 11 is increased in skin biopsies of SSc patients and correlates with fibrotic processes, including the transformation of FB into myofibroblasts (36, 37).

Similarly, the transcriptome of FB cultured with dcSSc-purified IgG from dcSSc ATA+ and dcSSc ATA− patients expressed a profibrotic profile in line with previous results in the literature (26). In our study, the DEP were enriched in ECM, regulation of the cytoskeleton or focal adhesion. In a previous transcriptomic analysis of inflammatory and fibroproliferative molecular subsets of SSc, skin genes from different datasets were also implied in cell adhesion and collagen binding (26, 38). Interestingly, genes that are usually associated with the fibro-proliferative subset of SSc like COL10A1, KIF20A, and TOP2A were also overexpressed by the FB exposed to dcSSc-purified IgG in our study (26, 28, 39).

Multi-omics analysis was performed in two ways: by restricting the analysis of the transcriptomics data to differentially expressed proteins, and by performing a global statistical analysis integrating proteomics and transcriptomics. Proteomic and transcriptomic variables, which had the highest contributions to the first components of the DIABLO model, were mostly overexpressed in the ATA+ group, which is consistent with single omics data. Interestingly, common variables between overexpressed variables in the ATA+ group in the integrative algorithm and proteins overexpressed in the ATA+ group in proteomic analysis were implied in myofibroblast signature and fibrosis features. Of these, the tubulin family proteins are polymerized into microtubules, a major part of the cytoskeleton. TUBB1 and TUBB3 are involved in the early stages of fibrosis (40). Transgelin is an actin cross-linking protein and is overexpressed in affected FB from SSc patients and upregulated in skin transcriptomics from dcSSc ATA+ patients (31, 41).

Our results revealed that IgG from SSc patients could induce the production of pro-fibrotic proteins and mRNA in normal FB, suggesting a potential pathogenic role. The mechanism of this pathogenicity remains unclear. Several hypotheses can be proposed: IgG binding on the surface of the FB either by Fab or Fc; or the internalization of IgG as it has been suggested in lupus with anti-DNA Aab. Recent data suggested that passive transfer of IgG from patients with fibromyalgia could induce symptoms and bind to the target; this reinforces the idea that IgG can play a direct pathogenic role (42).

Among all serotypes of SSc included in our study, the ATA+ group appeared highly different compared with HC and other subtypes, and we did not clearly identify ATA− and ACA+ groups. Among all the subtypes of ANA in SSc, ATA is the one whose pathogenicity is the most probable. TOPO-I antigen can bind heparan sulfate or chemokine receptor 7 at the FB membrane (43, 44).This binding could be amplified by ATA and recruit ATA, which could induce adhesion and activation of monocytes (44, 45). Anti-fibroblasts antibodies (AFA), which are capable of binding to FB, are found in up to 40% of SSc patients and are correlated with ATA positivity (46, 47). FB from SSc cultured in the presence of AFA-positive IgG enhance ECM degradation mRNA such as metalloproteinase-1 (MMP-1) (48). We found similar results in proteomics with COL1A1 overexpression in the presence of sera from SSc patients, whereas MMP-1 was overexpressed in the presence of purified IgG from SSc patients in the comparison of SSc sera vs. SSc-purified IgG. This result may point to a collagenolytic effect of SSc IgG. Nevertheless, MMP-1 and MMP-3 were not upregulated in the comparison of SSc purified IgG vs. HC-purified IgG and in ATA+ purified IgG vs. IgG purified HC. Thus, the overexpression of MMP-1 in the SSc IgG group may be due to the overexpression of COL1A1 in the SSc sera rather than to a collagenolytic effect of SSc IgG themselves. Moreover, AFA can upregulate FB secretion of proangiogenic chemokines such as CCl2 and CXCL8 (49). Since most patients positive for AFA are also positive for ATA, our results may highlight what was observed in studies focusing on AFA.

The strength of our study is the use of multi-omics analysis, which allowed us to study without any *a priori* assumption the contribution of SSc-purified IgG in ECM remodeling processes. These high-throughput omic technologies have shown promising results in SSc as intrinsic molecular subsets of patients can be identified mainly in skin biopsies (26). We developed here an omics approach to assess the potential of autoantibodies from the sera of patients, which is a highly accessible material in routine clinical practice. Age or disease duration may influence disease activity or ANA levels (17, 50, 51). In our cohort, there was no difference regarding age or disease duration between the groups.

The limitation of our study is the use of dermal normal FB instead of FB from SSc patients. FB from SSc patients can be intrinsically modified and there is a significant heterogeneity between FB in the same patient, precluding their use in this first concept study (52). A recent study using machine learning to analyze SSc skin gene expression showed different states of FB polarization according to disease duration and clinical improvement (53). The small number of patients led us to prioritize the correlations between transcriptomics and proteomics rather than the prediction of the group when using the DIABLO. However, the excellent correlation between transcriptomic and proteomic data and the natural separation of the groups led to very good discrimination of the three groups on the sample plots. The selected proteins and transcripts could be further investigated in a complementary study on more patients to assess their discriminative ability.

### Conclusion

Using a multi-omics approach, we identified that SSc-purified IgG can activate and induce ECM remodeling changes in the protein and mRNA expression profiles of dermal FB according to the serotype. IgG from dcSSc patients exhibited the most prominent ECM remodeling properties. Among these, ATA+ purified IgG showed a singular profile of protein expression, characterized by cell adhesion processes and autophagy. These results suggest that Aabs could play a pathophysiological role in SSc.

## Data Availability Statement

The datasets presented in this study can be found in online repositories. The names of the repositories and accession numbers can be found below: https://www.ncbi.nlm.nih.gov/, GSE178299; http://www.proteomexchange.org/, PXD025885.

## Ethics Statement

The studies involving human participants were reviewed and approved by CPP no. 2019-A01083-54, CNIL Data Protection Officer N°2018_82/DEC19-553, NCT04334031. The patients/participants provided their written informed consent to participate in this study.

## Author Contributions

All individuals listed as authors met the ICMJE guidelines for determining authorship. AC, SV, DL, SD and VS contributed to the conception and design of the study. AC, SV and LG performed experiments. FB and CR performed LC-MS/MS. JPM and MF performed transcriptomic analysis. CT and AF performed proteomics differential analyses and omics data integration under supervision of GM. AC performed the enrichment analysis. AC wrote the first draft of the manuscript. MF, GM, DL, SD and VS made major revisions to the manuscript. All authors revised, read and approved the submitted version.

## Funding

This study was partially found by the CSL Behring (grant award number RD1616). The funder was not involved in the study design, collection, analysis, interpretation of data, the writing of this article or the decision to submit it for publication.

## Conflict of Interest

DL reports grants from: GSK, Actelion, Boehringer Ingelheim, Takeda, CSL Behring, Biocryst. VS reports consultancies and speaking fees from Boehringer Ingelheim and Grifols (less than $10 000) and research support from Actelion, Grifols, GSK, Octapharma, Pfizer, Shire, outside the submitted work. GM works with former students, now employed by Diagrams Technologies and Genes Diffusion, outside of this present work.

The remaining authors declare that the research was conducted in the absence of any commercial or financial relationships that could be construed as a potential conflict of interest

## Publisher’s Note

All claims expressed in this article are solely those of the authors and do not necessarily represent those of their affiliated organizations, or those of the publisher, the editors and the reviewers. Any product that may be evaluated in this article, or claim that may be made by its manufacturer, is not guaranteed or endorsed by the publisher.

## Abbreviations

Aabs, autoantibodies; ATAs, anti-topoisomerase-I autoantibodies; ACAs, anti-centromere autoantibodies; dcSSc, diffuse cutaneous systemic sclerosis; DEGs, differential expressed genes; DIABLO, Data Integration Analysis for Biomarker discovery using Latent cOmponent; DEPs, differential expressed proteins; DNA, deoxyribonucleic acid; ECM, extracellular matrix; FB, fibroblast; FC, fold change; FCS, fetal calf serum; HCs, healthy controls; lcSSc, limited cutaneous SSc; LogFC, Log2 Fold Change; LC-MS/MS, liquid chromatography coupled with tandem mass spectrometry; NS, non stimulated; PCA, principal component analysis; SSc, systemic sclerosis; RNA, ribonucleic acid.
