# Supplementary material for: Effects of Immunoglobulins G From Systemic Sclerosis Patients in Normal Dermal Fibroblasts: A Multi-Omics Study
Source: Front Immunol. 2022 Jun 29;13:904631. doi: 10.3389/fimmu.2022.904631 (PMC9276964; doi:10.3389/fimmu.2022.904631)
Supplement: Supplementary file 1 [file DataSheet_1.docx]

**Supplemental data**

**Antibodies used for immunofluorescence assays**

|  | Antibodies references | Antibodies dilution |
| --- | --- | --- |
| Primary antibodies | S100A4 rabbit monoclonal IgG antibody, Invitrogen | 1/500 |
|  | RAB21 rabbit monoclonal IgG antibody, GeneTex |  |
| Secondary antibodies | Alexa Fluor 594 Donkey anti-rabbit IgG, antibody Biolegend | 1/1000 |

TABLES and FIGURES

| **Group** | **Sex** | **Age**  **(years)** | **Age at diagnosis**  **(years)** | **Disease duration (years, at inclusion)** | **mRSS (at study inclusion)** | **Organ involvement** | **ANA titer** | **ANA fluorescence** | **ENA type** | **IgG Titer (mg/mL)** | **Use of immunosuppressive agents** |
| --- | --- | --- | --- | --- | --- | --- | --- | --- | --- | --- | --- |
| [ATA+] | F | 27 | 23 | 5 | 15/51 | ILD | 1/1280 | Homogeneous /speckle/nucleolar | ATA | 8.08 | CYC/MMF |
|  | F | 50 | 50 | 1 | 13/51 | ILD | 1/1280 | Homogeneous/speckle/nucleolar | ATA | 11.19 | MMF |
|  | F | 46 | 27 | 16 | 13/51 | ILD | 1/1280 | Homogeneous/speckle/nucleolar | ATA | 12.77 | None |
|  | F | 53 | 35 | 15 | 9/51 | ILD | 1/640 | Homogeneous/speckle/ nucleolar | ATA | 7.42 | RTX |
|  | M | 48 | 46 | 2 | 23/51 | ILD  PAH | 1/680 | Homogeneous/speckle/ nucleolar | ATA | 13.52 | MMF |
| [ATA-] | F | 48 | 36 | 13 | 4/51 | DU | 1/320 | Without fluorescence | none | 18.51 | none |
|  | F | 60 | 52 | 9 | 3/51 | ILD | 1/80 | Nucleolar | PM-ScL | 15.10 | CYC/MMF |
|  | M | 36 | 36 | 1 | 19/51 | DU | >1/1280 | Nucleolar | Anti-fibrilarine | 11.58 | MTX |
|  | M | 69 | 63 | 6 | 5/51 | ILD | 1/320 | Nucleolar | Anti-PmSCL | 8.59 | CYC/MMF/MYF |
|  | M | 22 | 17 | 6 | 0/51 | ILD | 1/320 | Homogeneous | Without specificity | 19.90 | MTX |
| [ACA+] | F | 63 | 63 | 1 | 3/51 | DU | 1/1280 | Centromere | ACA | 9.38 | None |
|  | F | 50 | 50 | 1 | 2/51 | DU | 1/1280 | Centromere | ACA | 14.04 | None |
|  | F | 48 | 47 | 2 | 1/51 | DU | 1/1280 | Centromere | ACA | 13.47 | None |
|  | F | 64 | 59 | 6 | 7/51 | ILD | >1/1280 | Centromere | ACA | 9.43 | None |
|  | F | 50 | 42 | 6 | 0/51 | None | 1/680 | Centromere | ACA | 10.77 | None |
| [HC] | F | 45 |  |  |  |  |  |  |  | 8.77 |  |
|  | M | 26 |  |  |  |  |  |  |  | 14.26 |  |
|  | F | 27 |  |  |  |  |  |  |  | 14.64 |  |
|  | M | 58 |  |  |  |  |  |  |  | 9.31 |  |
|  | M | 30 |  |  |  |  |  |  |  | 13.71 |  |

**Supplemental table 1: Patients characteristics:** SSc: systemic sclerosis; [ATA+]: dcSSc anti-topoisomerase-I positive patients; [ATA-]: dcSSc anti-topoisomerase-I negative patients; [ACA+]: lcSSc anti-centromere positive patients; [HC]: healthy controls; ANA: antinuclear antibodies; ENA: extractable nuclear antigen; CYC: cyclophosphamide; MMF: mycophenolate mofetil; MTX: methotrexate; MYF: mycophenolic acid; RTX: rituximab; mRSS: modified Rodnan skin score; ILD: interstitial lung disease; PAH: pulmonary arterial hypertension; DU: digital ulcer.

**Supplementary table 2**

|  | [ATA+] | [ATA-] | [ACA+] | [HC] | p-value |
| --- | --- | --- | --- | --- | --- |
| Sex (female, %) | 80 | 40 | 100 | 40 | _ |
| Age at inclusion (years, mean ± SD) | 44.8 ± 10.3 | 47 ± 18.7 | 55 ± 7.8 | 37.2 ± 13.9 | 0.250 |
| Disease duration (years, mean ± SD) | 7.8 ± 7.2 | 7.0 ± 4.4 | 3.2 ± 2.6 | _ | 0.345 |

**Supplemental table 2:** [ATA+]: dcSSc anti-topoisomerase-I positive patients; [ATA-]: dcSSc anti-topoisomerase-I negative patients; [ACA+]: lcSSc anti-centromere positive patients; [HC]: healthy controls; SD: standard deviation.

**Supplementary table 3**

|  | Patient 1 | Patient 2 |
| --- | --- | --- |
| IL-10 (pg/mL) | <1.16 | <1.16 |
| IL-1β (pg/mL) | 1.87 | 2.73 |
| IL-6 (pg/mL) | <0.56 | 1.50 |
| TNF-α (pg/mL) | 1.22 | 0.982 |

**Supplemental figure 3:** Cytokines assays in purified IgG from two dcSSc ATA+ patients.


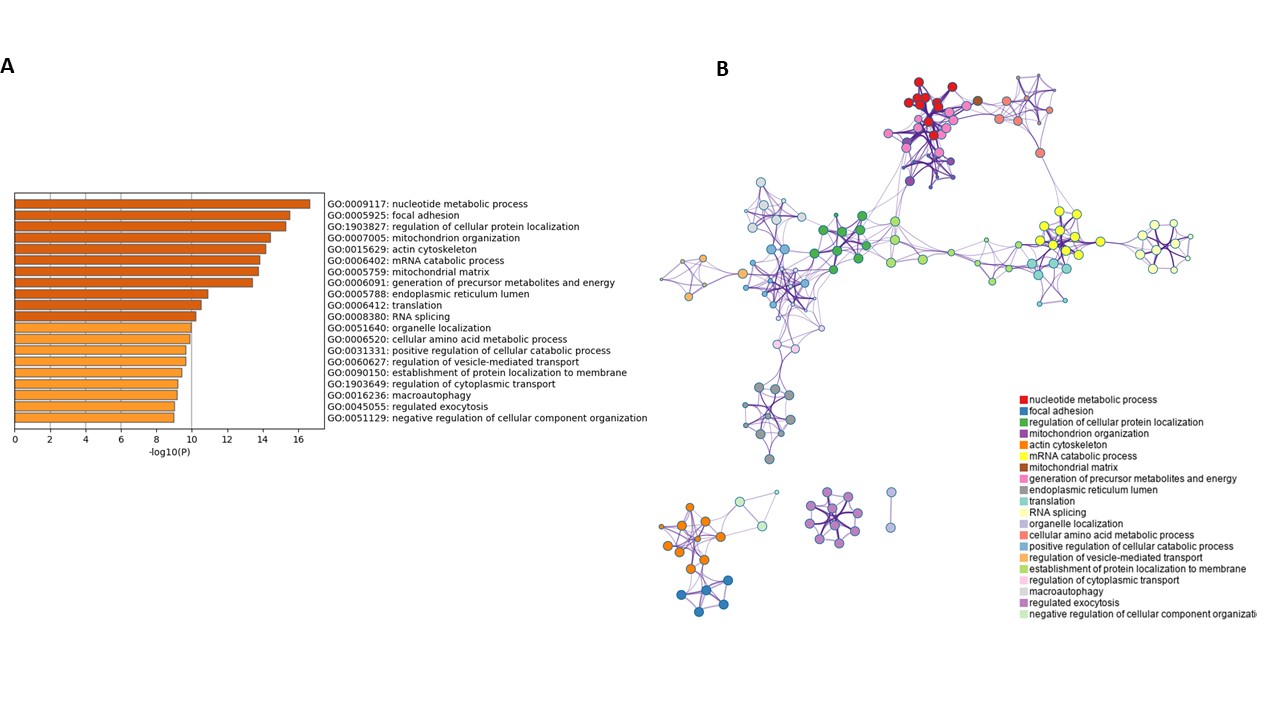


**Supplemental figure 1:** (A) and (B) Enriched GO terms in cluster 1 according to upregulated proteins, analysis done with Metascape. *GO terms: genes ontology terms.*


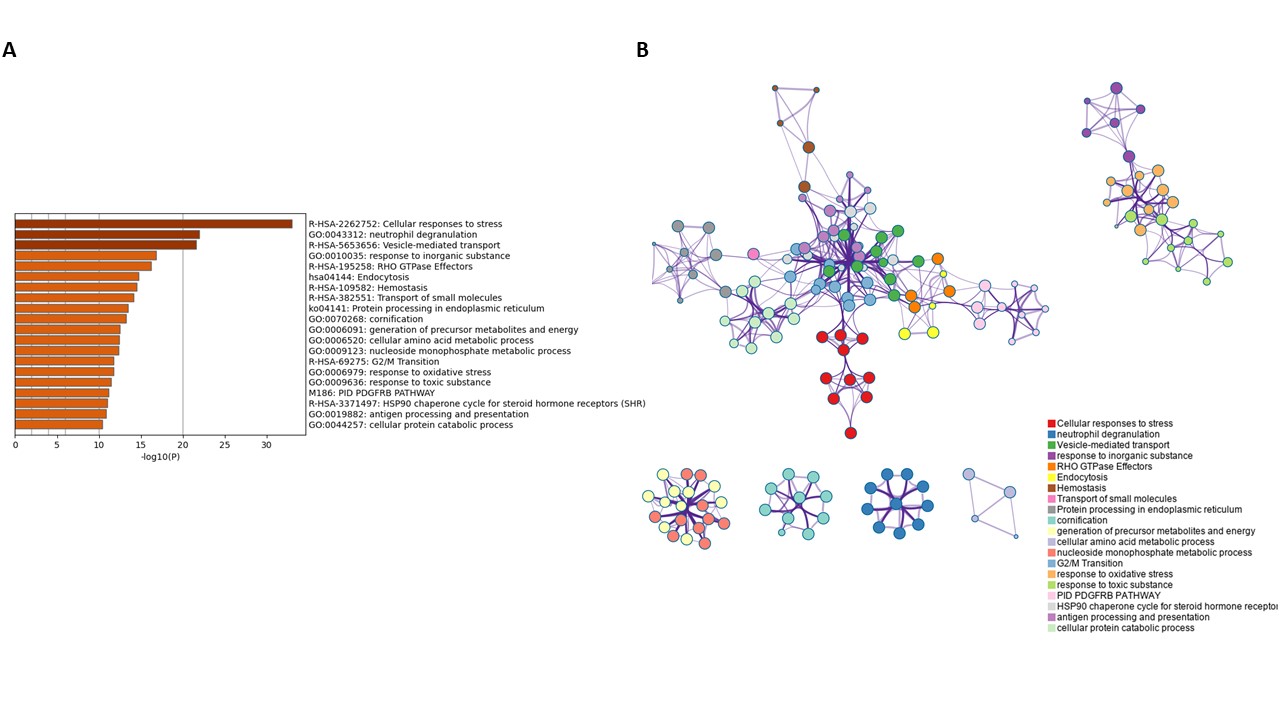


**Supplemental figure 2:** (A) and (B) Enriched GO terms in cluster 2 according to upregulated proteins, analysis done with Metascape. *GO terms: genes ontology terms.*


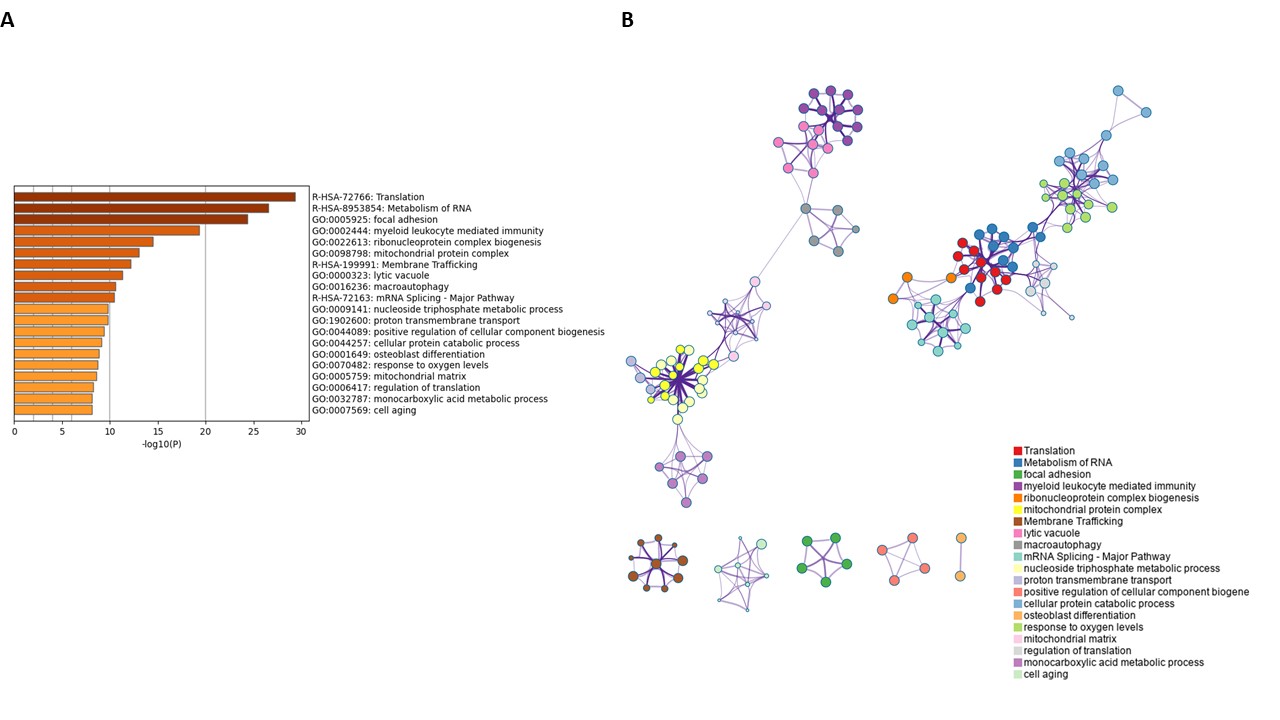


**Supplemental figure 3:** (A) and (B) Enriched GO terms in cluster 3 according to upregulated proteins, analysis done with Metascape. *GO terms: genes ontology terms.*


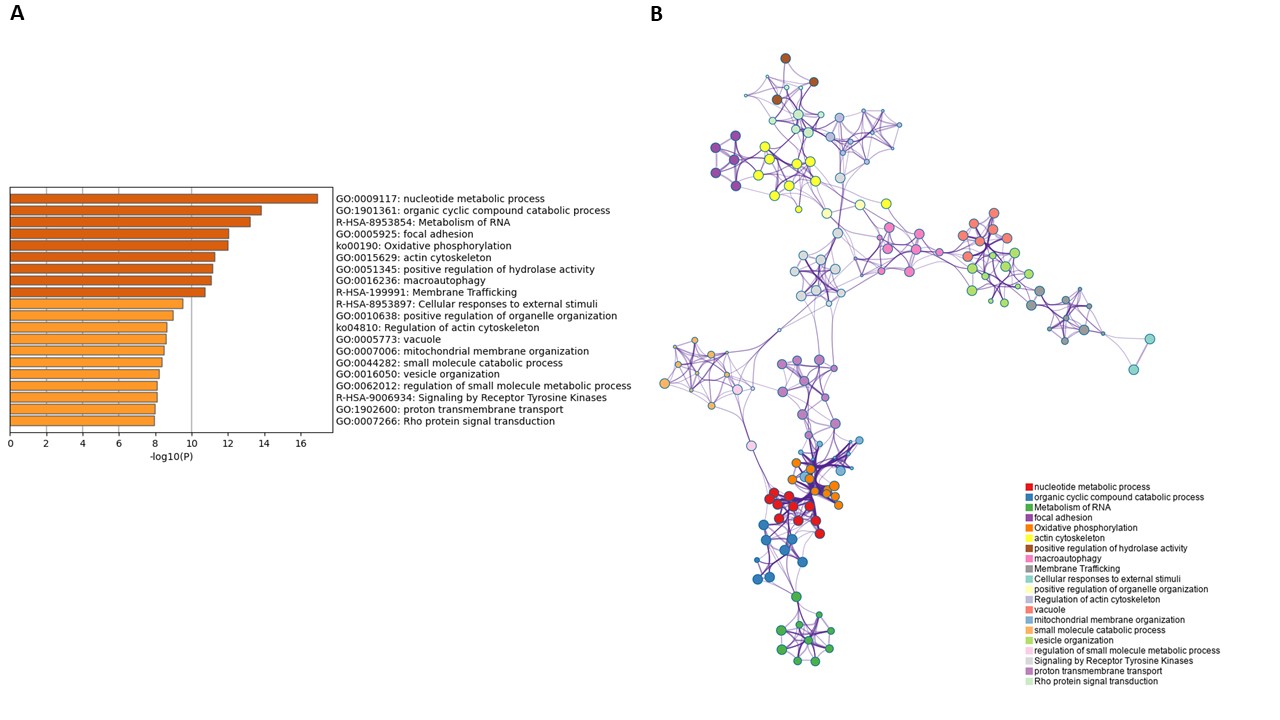


**Supplemental figure 4:** (A) and (B) Enriched GO terms in cluster 4 according to upregulated proteins, analysis done with Metascape. *GO terms: genes ontology terms.*


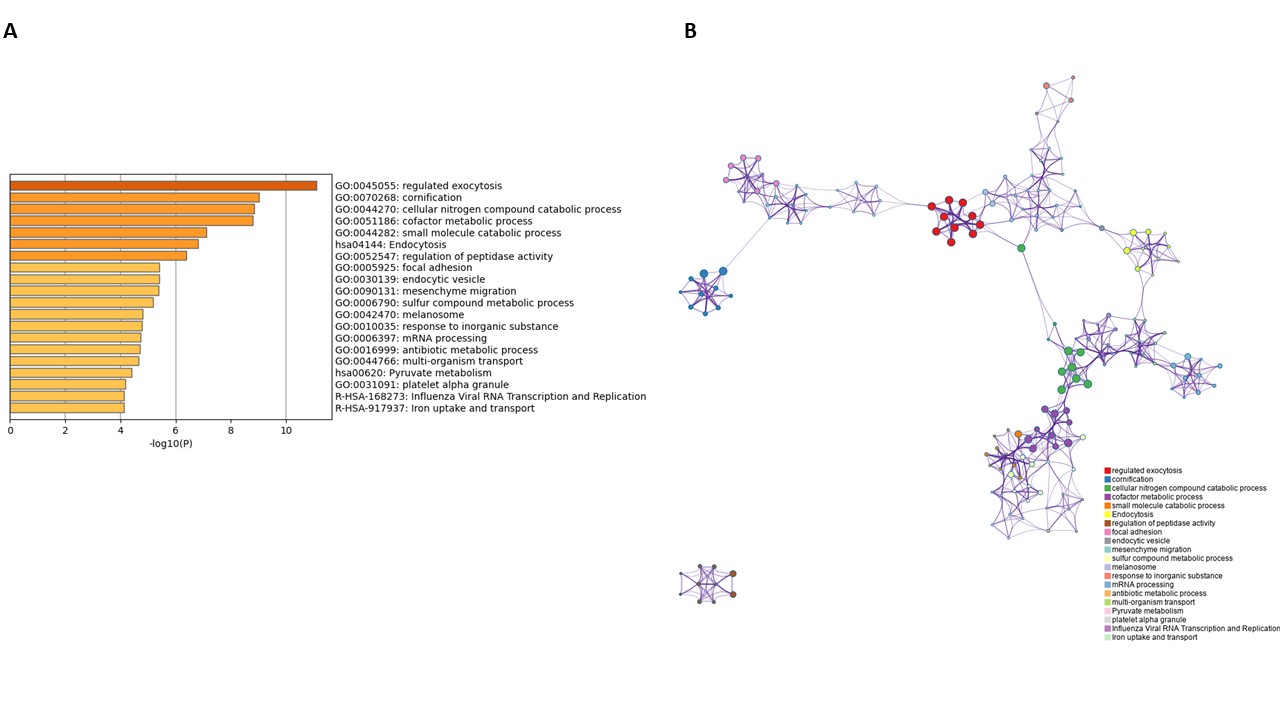


**Supplemental figure 5:** (A) and (B) Enriched GO terms in cluster 5 according to upregulated proteins, analysis done with Metascape. *GO terms: genes ontology terms*


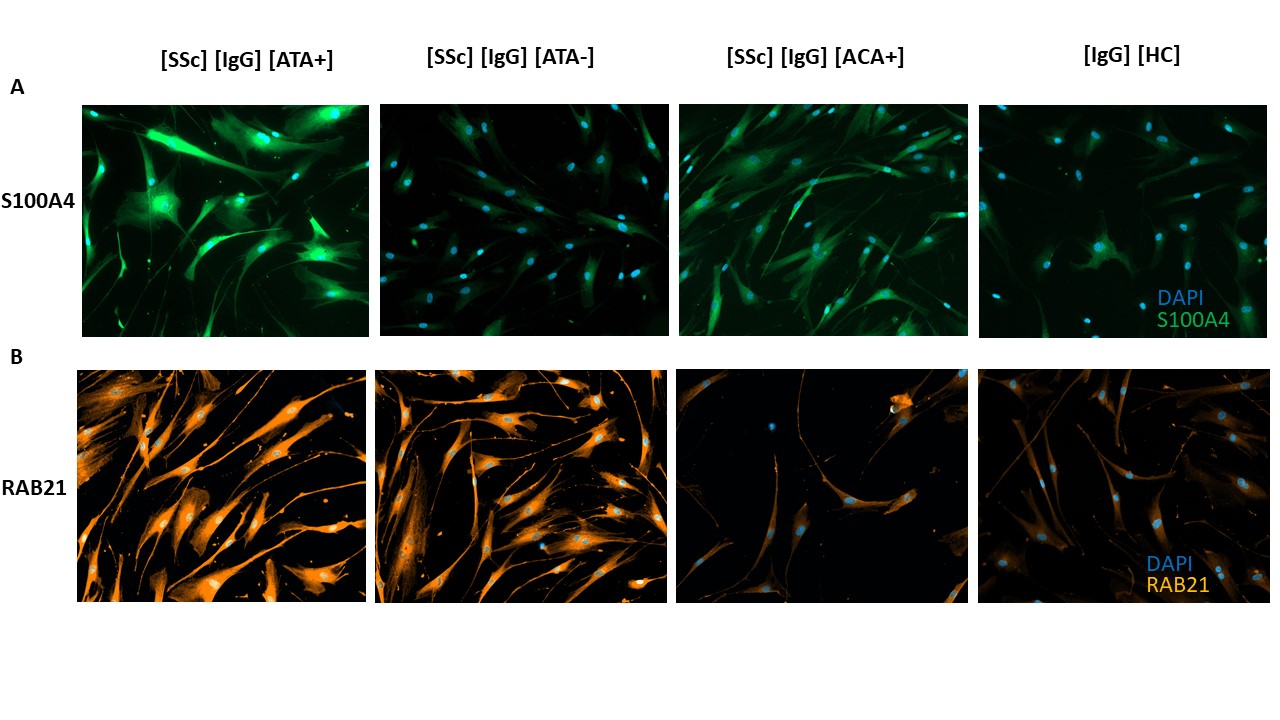


**Supplemental figure 6:** FB immunofluorescence assays; (A) immunofluorescence staining of proteins S100 A4 on FB in the presence of purified IgG from [SSc] [IgG] [ATA+], [SSc] [IgG] [ATA-], [SSc] [IgG] [ACA+] and [HC] [IgG]; (B) immunofluorescence staining of protein RAB21 on FB in the presence of purified IgG from [SSc] [IgG] [ATA+], [SSc] [IgG][ATA-], [SSc] [IgG] [ACA+] and [HC] [IgG]; magnification X 20

*[SSc] [IgG] [ATA+]: IgG from dcSSc anti-topoisomerase-I positive patients; [SSc] [IgG] [ATA-]: IgG from dcSSc anti-topoisomerase-I negative patients; [SSc] [IgG] [ACA+]: IgG from dcSSc anti-topoisomerase-I negative patients; [HC] [IgG]: IgG from healthy controls.*


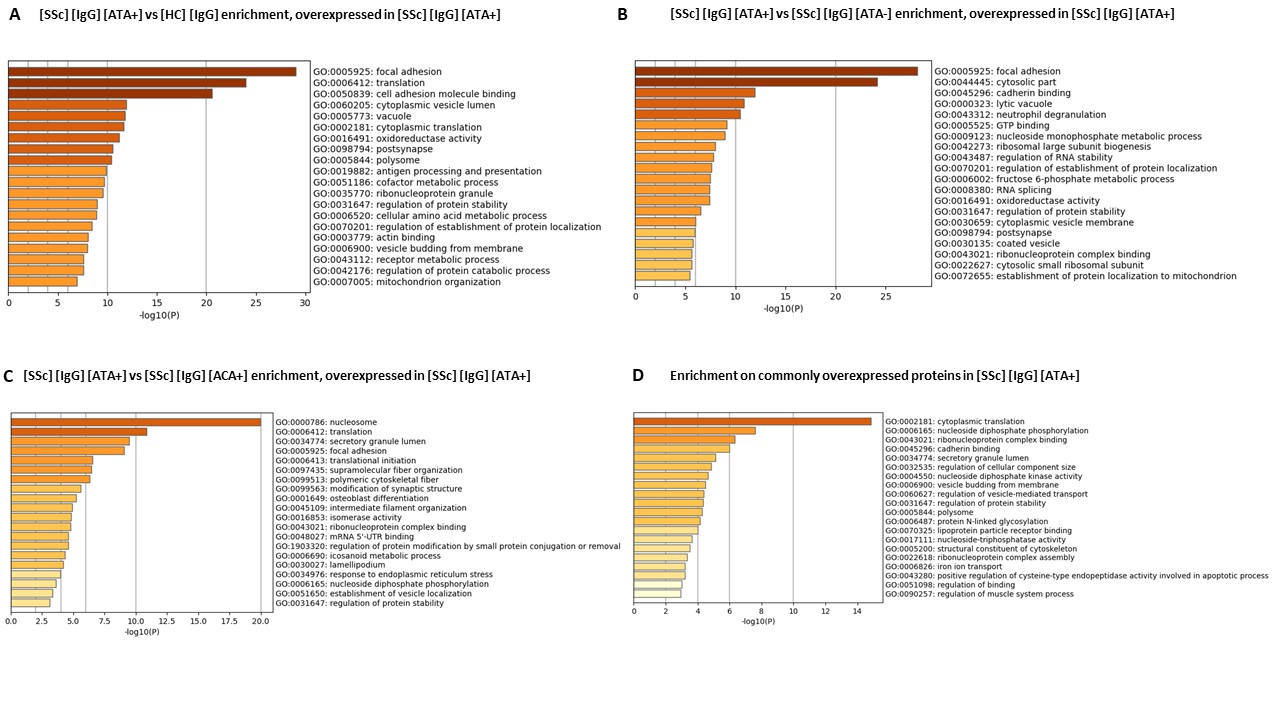
**Supplemental figure 7:** Enrichment analysis in ATA+ group; (A) Enriched GO terms in the comparison [SSc] [IgG] [ATA+] vs [HC] [IgG] according to upregulated proteins (overexpressed in [SSc] [IgG] [ATA+]); (B) Enriched GO terms in the comparison [SSc] [IgG] [ATA+] vs [SSc] [IgG] [ATA-] according to upregulated proteins (overexpressed in [SSc] [IgG] [ATA+]); (C) Enriched GO terms in the comparison [SSc] [IgG] [ATA+] vs [SSc] [IgG] [ACA+] according to upregulated proteins (overexpressed in [SSc] [IgG] [ATA+]); (D) Enriched GO terms in proteins commonly overexpressed in [SSc] [IgG] [ATA+]).

*Enriched GO terms done with Metascape. GO terms: gene ontology terms; [SSc] [IgG] [ATA+]: IgG from dcSSc anti-topoisomerase-I positive patients; [SSc] [IgG] [ATA-]: IgG from dcSSc anti-topoisomerase-I negative patients; [SSc] [IgG] [ACA+]: IgG from dcSSc anti-topoisomerase-I negative patients; [HC] [IgG]: IgG from healthy controls.*


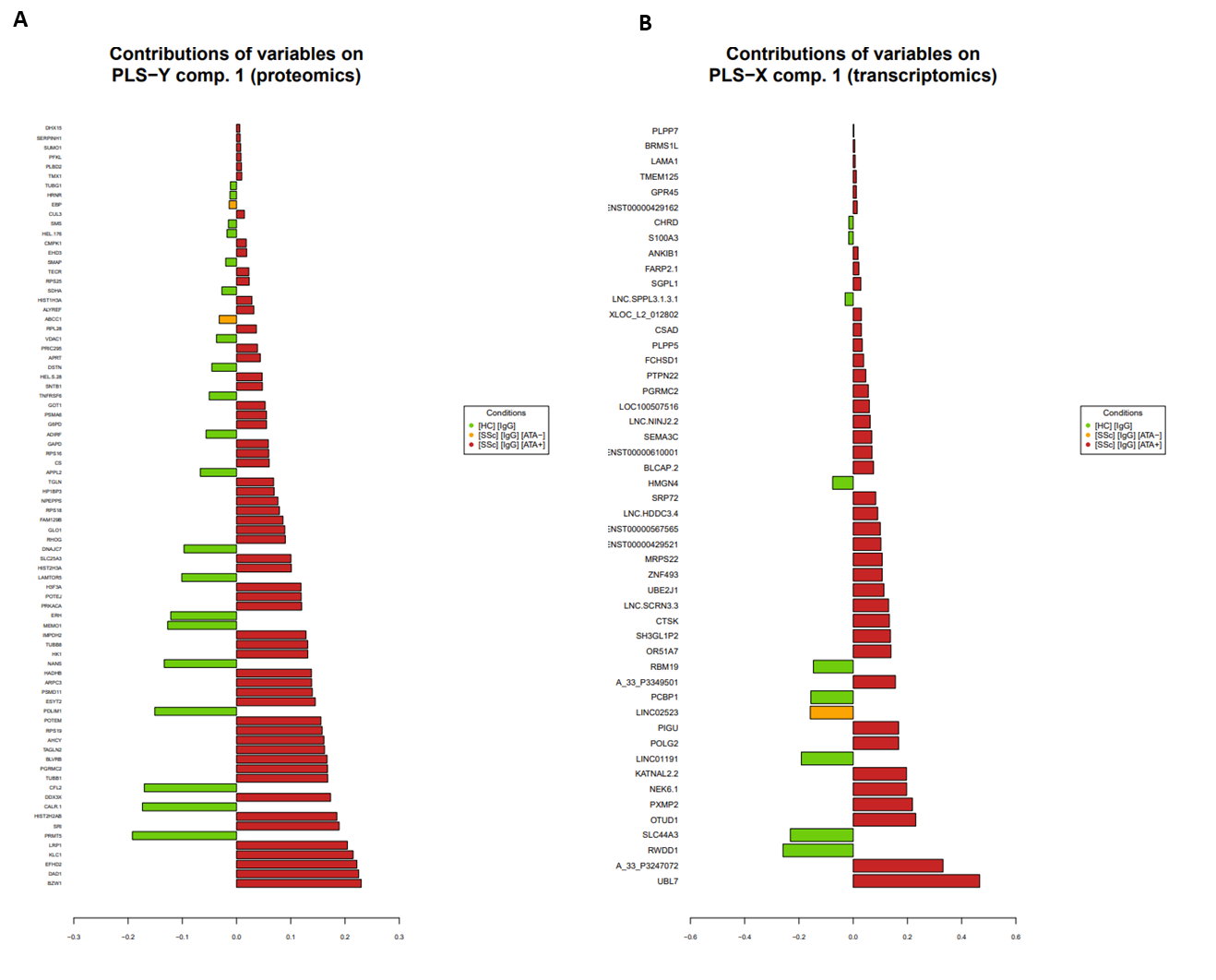


**Supplemental figure 8:** Proteomics and transcriptomics integration. Most contributing variables on first latent component (PLS-X comp.1) for (A) proteomics and (B) transcriptomics data.

*[SSc] [IgG] [ATA+]: IgG from dcSSc anti-topoisomerase-I positive patients; [SSc] [IgG] [ATA-]: IgG from dcSSc anti-topoisomerase-I negative patients; [SSc] [IgG] [ACA+]: IgG from dcSSc anti-topoisomerase-I negative patients; [HC] [IgG]: IgG from healthy controls.*


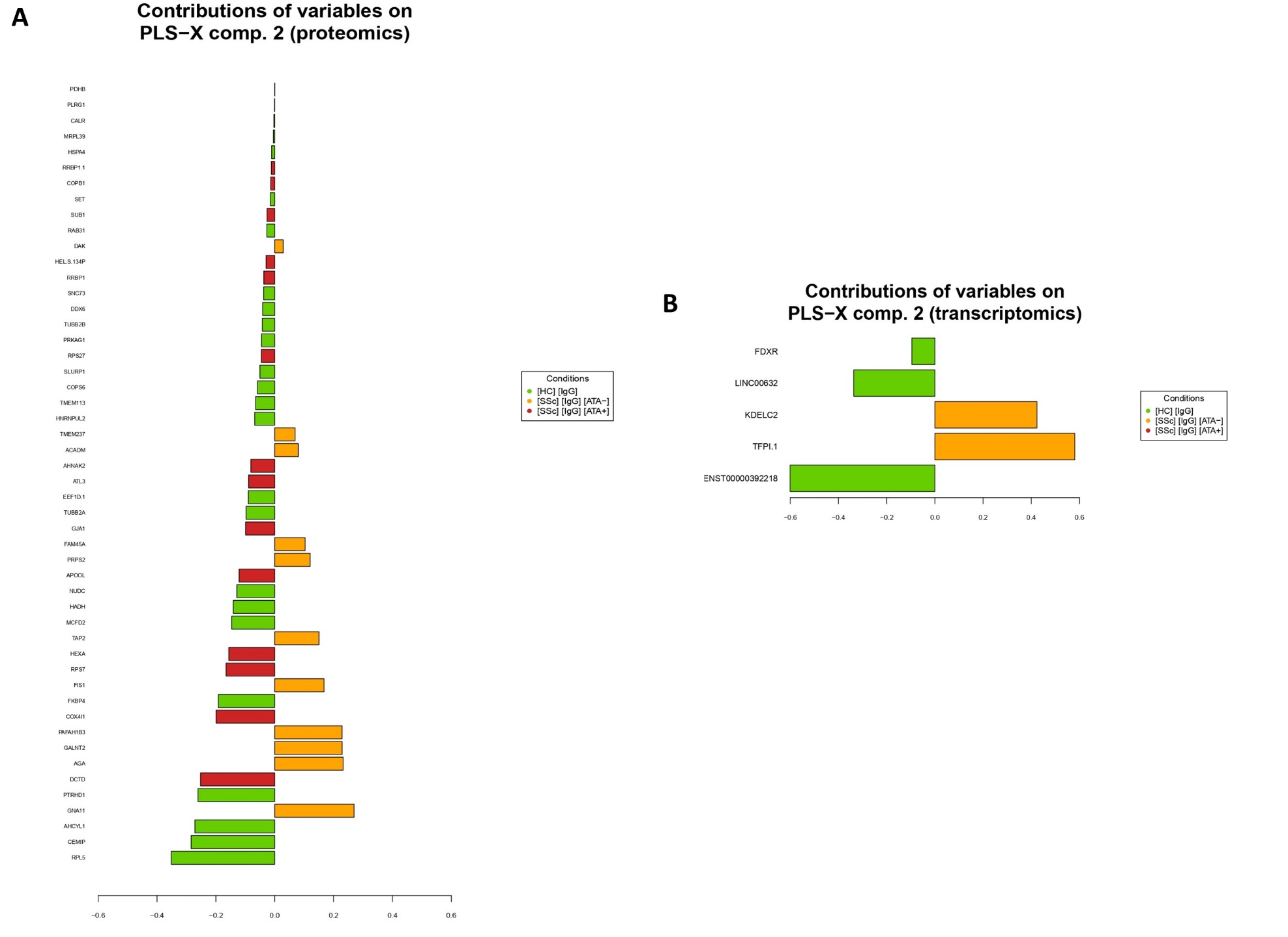


**Supplemental figure 9:** Proteomics and transcriptomics integration. Most contributing variables on second latent component (PLS-X comp.2) for (A) proteomics and (B) transcriptomics data.

*[SSc] [IgG] [ATA+]: IgG from dcSSc anti-topoisomerase-I positive patients; [SSc] [IgG] [ATA-]: IgG from dcSSc anti-topoisomerase-I negative patients; [SSc] [IgG] [ACA+]: IgG from dcSSc anti-topoisomerase-I negative patients; [HC] [IgG]: IgG from healthy controls.*


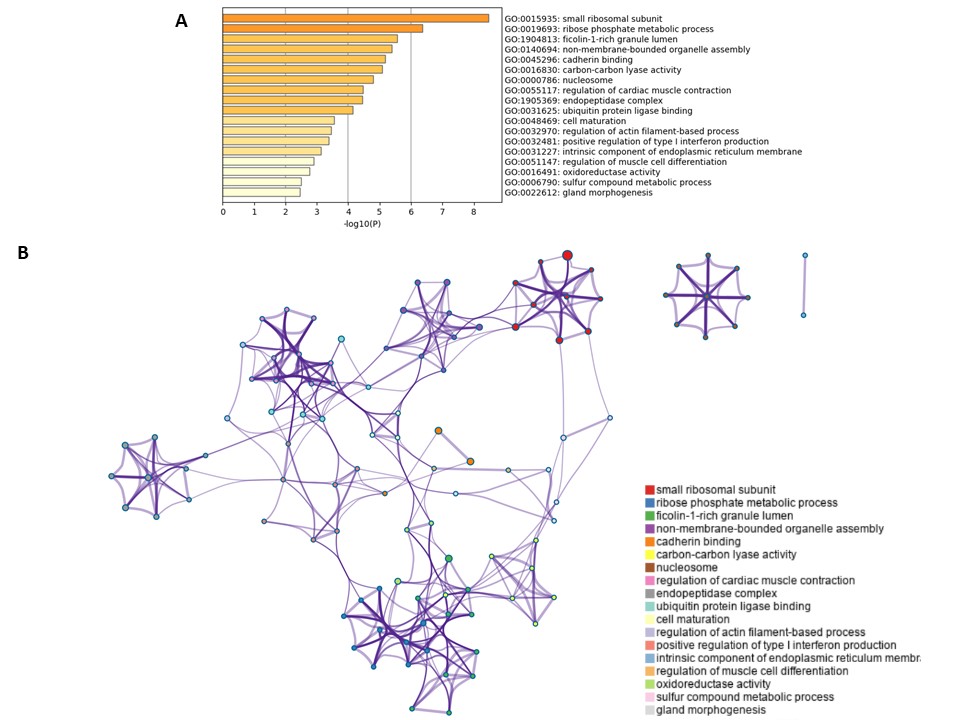


**Supplemental figure 10:** Cluster 1 of Omics integration enrichment; (B) and (C) Enriched GO terms in cluster 1 according to upregulated proteins, analysis done with Metascape.
