# Supplementary figures and images for: Effects of Immunoglobulins G From Systemic Sclerosis Patients in Normal Dermal Fibroblasts: A Multi-Omics Study
Source: Front Immunol. 2022 Jun 29;13:904631. doi: 10.3389/fimmu.2022.904631 (PMC9276964; doi:10.3389/fimmu.2022.904631)

## Slide 1
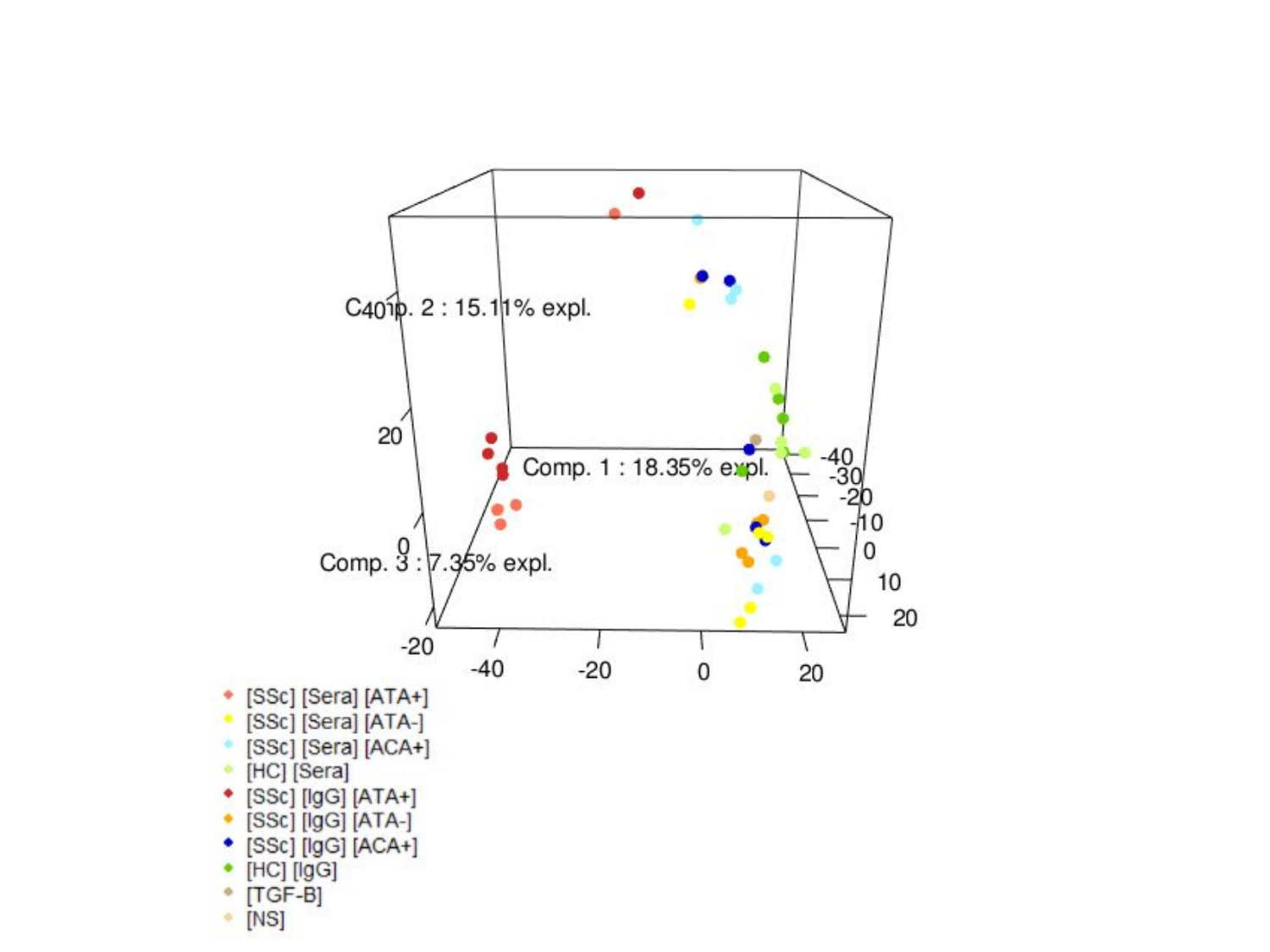

Supplement: Supplementary file 2 [file Presentation_1.pptx]
